# Porous Waste Glass for Lead Removal in Packed Bed Columns and Reuse in Cement Conglomerates

**DOI:** 10.3390/ma12010094

**Published:** 2018-12-28

**Authors:** Andrea Petrella, Danilo Spasiano, Marco Race, Vito Rizzi, Pinalysa Cosma, Stefania Liuzzi, Nicoletta De Vietro

**Affiliations:** 1 Dipartimento di Ingegneria Civile, Ambientale, Edile, del Territorio e di Chimica, Politecnico di Bari, Via E. Orabona, 4, 70125 Bari, Italy; danilo.spasiano@poliba.it; 2 Dipartimento di Ingegneria Civile e Meccanica, Università di Cassino e del Lazio Meridionale, Via Di Biasio 43, 03043 Cassino, Italy; marco.race@unina.it; 3 Dipartimento di Chimica, Università degli Studi di Bari “Aldo Moro”, Via E. Orabona, 4, 70125 Bari, Italy; vito.rizzi@uniba.it (V.R.); pinalysa.cosma@uniba.it (P.C.); 4 Dipartimento di Scienze dell’Ingegneria Civile e dell’Architettura, Politecnico di Bari, Via E. Orabona, 4, 70125 Bari, Italy; stefania.liuzzi@poliba.it; 5 Istituto di Nanotecnologia (Nanotec), Consiglio Nazionale delle Ricerche (CNR), c/o Dipartimento di Chimica, Università degli Studi di Bari “Aldo Moro”, Via E. Orabona, 4, 70125 Bari, Italy; nicoletta.devietro@uniba.it

**Keywords:** lead ion, recycled waste porous glass, column experiments, ion exchange, film diffusion control, cement mortar

## Abstract

A porous waste glass (RWPG = recycled waste porous glass) was used in wastewater treatments for the removal of lead ions from single, binary, and ternary metal solutions (with cadmium and nickel ions). Experiments were performed in columns (30 cm^3^, 10 g) filled with 0.5–1 mm beads till complete glass exhaustion (breakthrough). In the case of single and binary solutions, the columns were percolated at 0.2 Lh^−1^ (2 mg Me^+2^ L^−1^); in the case of ternary solutions, the columns were percolated at 0.15–0.4 Lh^−1^ (2 mg Me^2+^ L^−1^) and with 2–5 mg Me^2+^ L^−1^ influent concentration (0.2 Lh^−1^). Lead ions were removed mainly by ion exchange and also by adsorption. From a kinetic point of view, the rate controlling step of the process was the interdiffusion of the lead ions in the Nernst stationary liquid film around the sorbent. The uptake of the metals and the glass selectivity were confirmed by Energy Dispersive X-ray spectroscopy (EDX) analysis. After lead retention process, glass beads were reused as lightweight aggregates for thermal insulating and environmental safe mortars.

## 1. Introduction

A large number of chemicals are today released in water [1,2,3], land [4,5], or air [6,7], with severe impacts on the environment and consequently on human health. Environmental remediation is based on the removal of these contaminants from air [8,9], soil [10,11], sediment, groundwater, and surface water [12,13,14,15] which are carried out with specific technological approaches. 

Heavy metals (HM) are inorganic biopersistent pollutants, potentially hazardous to animals and human health [16,17,18,19], and derived from mining, leather tanning, industrial wastes, metals smelting, batteries, paints, metal finishing operations, vehicle emissions, fertilizers, etc. [20,21]. Heavy metal contamination generates severe adverse health effects; for this reason, the risks related to every environmental compartment need to be overcome [19,22,23,24,25]. Among heavy metals, lead is a particularly dangerous pollutant with a large presence in the environment due to a wide range of applications. It can accumulate in water and soil organisms but also in individuals and in the entire food chains, with severe risks associated to the toxicity of the lead compounds [26,27].

In the case of water pollution, the most used approaches to remove heavy metals from wastewaters are adsorption by activated carbon [28,29] and ion exchange by synthetic resins/zeolites [30,31,32,33]. Generally, low cost sorbents can be also assumed for these treatments because abundant in nature or deriving from agricultural and municipal/industrial wastes as in the case of recycled porous glass [23,34,35,36,37]. Specifically, the use of glass as byproduct form household and industrial wastes is fundamental for the improvement of the environmental sustainability because glass is a biopersistent material that otherwise would be landfilled [38,39,40]. Moreover, recycling is an important task because less energy is required for this operation as opposite to the energy required for manufacturing glass from sand, soda, and lime. In addition, glass can be easily reused without modification of the peculiar characteristics; accordingly, interest in the potential recovery and reuse of this novel material has recently increased since this material is characterized by durability, safety, excellent hardness, abrasion resistance, and negligible water absorption [41,42,43,44]. 

Recycled Waste Porous Glass (RWPG) used in the present research is obtained after raw material heating at 900 °C and successively introduction of porosizing agents in the mixture as calcium carbonate. The final structure of this secondary raw material is porous and light, features exploitable in the construction industry (lightweight concrete and mortar aggregates, thermal insulating materials, and road paving filler) [45,46,47].

In the present work, RWPG columns are used for the removal of Pb^+2^ ions in single, binary, and multimetal solutions (with cadmium and nickel ions). Synthetic lead ion concentration was chosen in a range comparable to the industrial practice (battery production, fertilizers, mining, and metallic industries), which is ~2–5 mg L^−1^ [48,49]. The metal retention phenomena were thermodynamically and kinetically evaluated at room temperature, different flow rates and influent concentration, and a structural characterization of the material after the sorption process was carried out. The advantage of the use of this type of cheap sorbent is the re-employment of the metals exhausted glass directly as lightweight aggregate with no problems of hazardous sludge production because the original properties of this secondary raw material (thermal insulation) were preserved. For this reason, RWPG beads were finally encapsulated in cement conglomerates in order to prevent any release of metals in the environment. The resulting lightweight conglomerates may be applied as constructive elements due to the low thermal conductivity. 

The approach adopted in this study is in agreement with the principles of the circular economy, since it contributes to the sustainable water treatment and raw material reuse efforts, as reported in many other studies on waste and wastewater treatments [50,51,52,53,54] 

## 2. Experimental Section

### 2.1. Materials and Methods

RWPG is a typical sodium calcium silicate glass (chemical composition: 71% SiO_2_, 9% CaO, 14% Na_2_O, 3% Al_2_O_3_, 2% MgO, and 1% K_2_O) derived from municipal and industrial solid wastes. At first, the raw materials were cleaned and crushed, successively a porosizing agent was added to the molten glass at 900–1300 °C. The final material shows a specific weight in the range of 200 to 900 kg m^−3^ and a controlled porosity. 

BET (Brunauer-Emmett-Teller) determinations (adsorption–desorption N_2_ isotherms at 77 K) of the glass beads were carried out by an Autosorb IQ Chemi TCD instrument (Quantachrome Instruments, Boynton Beach, FL, USA) at a relative pressure of 6.58 × 10^−5^ 1/Torr. Experimental results were processed utilizing the ASiQwin software (ver 4.0, Boynton Beach, FL, USA) and, in a specific way, the BJH (Barrett, Joyner, Halenda) pore size distribution method was employed to determinate the surface area and the volume and radius (Dv(r)) of the porous. The glass samples were outgassed prior for 3 h at 373 K. 

Scanning Electron Microscopy (SEM) observations were obtained with a Zeiss scanning electron microscope model EVO50XVP (Carl Zeiss Microscopy GmbH, Jena, Germany). The samples were applied to aluminum stubs and a carbon film was deposited by sputtering (Edwards Auto 306 thermal evaporator, Edwards, Burgess Hill, UK). Back-scattered electron (BSE) images were obtained at 15 kV accelerating potential and 500 pA probe current. EDX analysis of the metals laden RWPG was obtained by an electron microscope FESEM-EDX Carl Zeiss Sigma 300 VP (Carl Zeiss Microscopy GmbH, Jena, Germany). 

### 2.2. Column Tests

**Figure 1:**
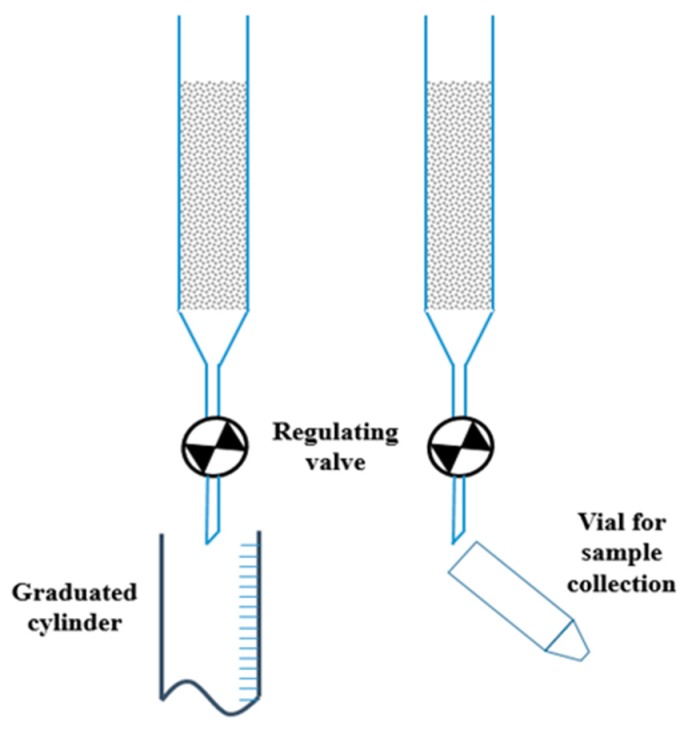
Column experiment and sample collection.

Solutions were prepared in distilled water (pH = 6) from reactive grade Pb(NO_3_)_2_, Cd(NO_3_)_2_·4H_2_O, and Ni(NO_3_)_2_·7H_2_O (Carlo Erba, Milan, Italy). Experiments were carried out in packed bed columns which allowed to obtain metals breakthrough curves which represent the relative effluent concentration (C/C_0_) vs. time (t) or vs. the effluent Bed Volumes (BV = V/V_0_). In particular, the regulation of the valve located at the bottom of the column allowed to work with different flow rates (Figure 1). During the experiments, the effluents were collected in a graduated cylinder to measure the volume treated or in a vial (10 ml) when samples were withdrawn. Complete exhaustion of the glass material (column breakthrough) corresponded to the effluent concentration equal to the influent concentration, and the breakpoint corresponded to the minimum retention capacity of the sorbent toward the metals.

The retention capacities (q_exp_) were obtained by dividing the upper area of the breakthrough curves with the amount of glass used in the packed bed column. 

Columns were percolated with single, binary, and ternary solutions of lead, cadmium, and nickel ions and the experiments were performed at room temperature, at the same bead size range (0.5–1 mm), and glass volume and dosage (30 cm^3^, 10 g). 

At first, the sorbent was percolated with single metal solutions (2 mg Me^+2^ L^−1^) at 0.2 Lh^−1^. Successively, the sorbent was percolated with binary metal solutions (2 mg Me^+2^ L^−1^) at 0.2 Lh^−1^. Finally, the sorbent was percolated with ternary metal solutions at flow rates in the range of 0.15 to 0.4 Lh^−1^ (2 mg Me^+2^ L^−1^) and at different metal concentrations (2–5 mg Me^+2^ L^−1^) at 0.2 Lh^−1^. Table 1 represents a summary of the tests performed in the present research. All experimental runs were performed in triplicate and each sample was analyzed three times. An Ati Unicam 929 Model flame atomic absorption spectrophotometer (FAAS) from UNICAM, Cambridge, England, equipped with an ATI UNICAM hollow cathode lamp was used for the metals determinations. A mixture of acetylene as a fuel, air as an oxidizing agent, and a laminar flow burner was used.

Kinetic observations, represented by time-dependent breakthrough curves (C/C_0_ vs. t), were also carried out together with thermodynamic observations (C/C_0_ vs. BV). As reported elsewhere [36], the sorption kinetics were considered under the mass transfer phenomena control and the rate determining step of the process was represented by the film diffusion control (resistance to ion interdiffusion into the liquid–solid Nernst interface) because of the prevalence of the functional groups on the surface of the sorbent [36].

Specifically, the kinetic equation was obtained after integration of the second Fick’s law together with the mass/charge balance in the film. Film thickness was assumed as the spatial coordinate, moreover it was assumed the infinite solution volume condition, i.e., exceeding amount of RWPG particles respect to the entering ions (C_RWPG_V_RWPG_ >> C_M_^++^V_M_^++^) [36]:

U(t) = 3DS/2r_0_δ(C_0_ − C_e_) (1)

D is the ions interdiffusion coefficient, S is the exposed surface area, r_0_ is the particle radius, δ is the film thickness of the Nernst liquid film around the particle, C_0_ is the bulk concentration of the entering species, C_e_ is the concentration of the species at the liquid–solid interface, U(t) is the fractional attainment of equilibrium. The half time of reaction (t_0.5_) is represented by Equation (2):

t_0.5_ = 1.33r_0_δ/DC_0_S (2)

Equation (2) is obtained when the concentration of the entering ion is reduced by half respect to the bulk ion concentration and shows that t_0.5_ is directly proportional to the particle radius (r_0_) and film thickness (δ) and inversely proportional to the bulk ion concentration (C_0_). 

### 2.3. Applications of RWPG Beads After Sorption

Pb^+2^ exhausted glass beads were finally used as aggregates for the preparation of cement composites. Class II CEM A-LL, 42.5R cement, from Buzzi Unicem, Barletta, Italy [55], was used as ligand. To the purpose, 450 g of cement, 225 cm^3^ of water, and 400 cm^3^ of RWPG were mixed (RWPG sample) [56]. Cube specimens (4 × 4 × 4 cm) were prepared, cured for 28 days (RH > 90%), and successively submitted to the jar test (Vittadini, Aqua, Milan, Italy) to evaluate the potential release of the metal species in water, which was carried out after filtration and analysis of the supernatant solution [57] after 24 h of immersion in distilled water. After the jar test, other cement conglomerates were prepared with replacement of the glass aggregate. Specifically, a porous alumosilicate as perlite (0–1 mm bead size range) was introduced in partial replacement of glass in order to give to the resulting mortars higher mechanical resistances. In this case, 450 g of cement, 225 cm^3^ of water, 200 cm^3^ of RWPG, and 200 cm^3^ of perlite were used (perl/RWPG sample). A sample based on bare perlite was also prepared, constant cement and water dosage (perlite sample). A normalized sand-based mortar was prepared as reference sample [56]. Compression strengths were obtained on twelve semiprisms (loading rate in the range of 2400 ± 200 N/s), deriving from the flexural tests carried out on six prisms (40 × 40 × 160 mm) (loading rate in the range of 50 ± 10 N/s) [56]. A MATEST device, Milan, Italy, was employed for the mechanical tests.

Thermal conductivity (λ) measurements were also carried out and a Mod. ISOMET 2104 device, from Applied Precision Ltd (Bratislava, Slovakia) was used. Specifically, a heating probe applied to the surface of a cylindrical sample (diameter = 100 mm; height = 50 mm, 28 days cured) generated a constant thermal flow which allowed to obtain the thermal conductivity after comparison of the experimental temperature values with the analytical solution of the heat conduction equation [58].

## 3. Results and Discussion

**Figure 2:**
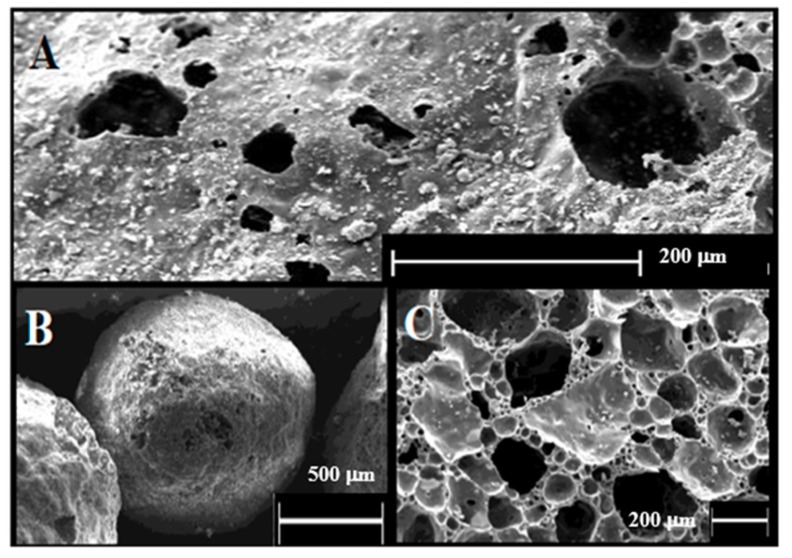
SEM back-scattered electron images of (**A**) the surface of a RWPG grain, of (**B**) a typical glass bead, and of (**C**) the internal texture of a grain.

The Scanning Electron Micrographs (SEMs) of a RWPG bead are shown in Figure 2. A large external (Figure 2A) and internal porosity (Figure 2C, section of the glass particle) can be observed. From BET determinations (adsorption–desorption N_2_ isotherms at 77 K), RWPG is a mesoporous material with pore diameters ranging from 20 to 500 Å, average pore radius (Dv(r)) in the range of 15.5 Å, and BET total surface area exceeding 2 m^2^ g^−1^. Referring to the pores, the total volume and surface area were in the range of 7.0 mm^3^ g^−1^ and 1.0 m^2^ g^−1^, respectively.

**Figure 3:**
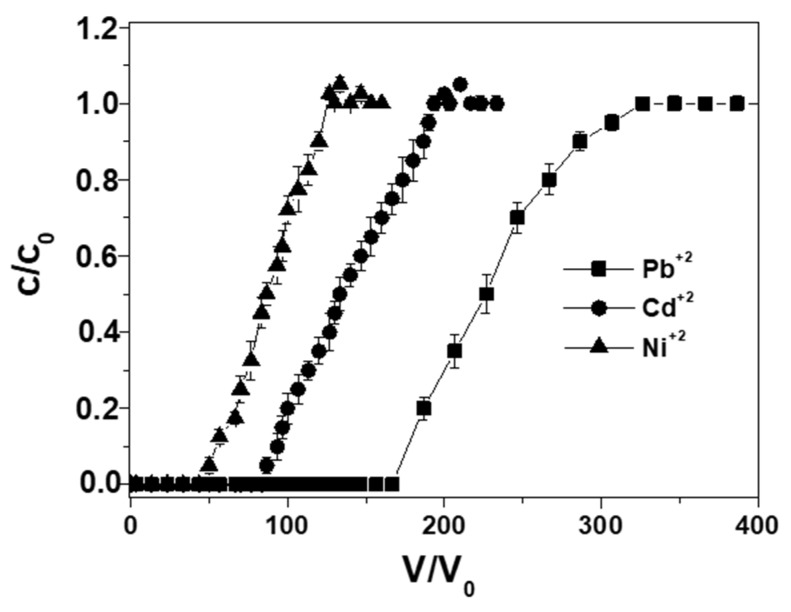
Breakthrough curves in distilled water for single ion solutions of lead, cadmium, and nickel ions (0.2 Lh^−1^ flow rate, 2 mg Me^+2^ L^−1^, 10 g RWPG, 0.5–1 mm bead size range, pH = 6, and T = 298 K).

Figure 3 shows a comparison of the breakthrough curves among single ion solutions (2 mg Me^+2^ L^−1^) at constant flow rate (0.2 Lh^−1^) and particle dosage (10 g). The overall retention capacities were 1.45, 0.85, and 0.50 mg·g_RWPG_^−1^ and breakpoints were 160, 80, and 40 BV for Pb^+2^, Cd^+2^ and Ni^+2^, respectively (Table 2). 

**Figure 4:**
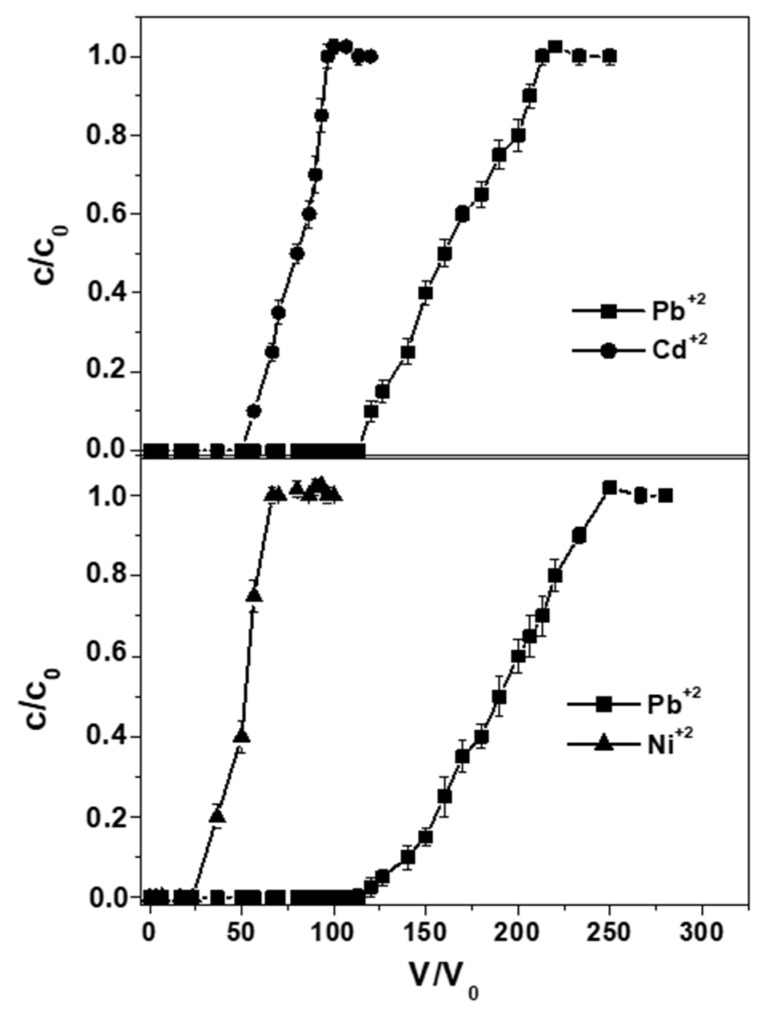
Breakthrough curves in distilled water of lead/cadmium and lead/nickel binary solutions (0.2 Lh^−1^ flow rate, 2 mg Me^+2^ L^−1^, 10 g RWPG, 0.5-1 mm bead size range, pH = 6, and T = 298 K).

Table 3 and Figure 4 show a comparison of the metals overall capacities for tests carried out in binary solutions (2 mg Me^+2^ L^−1^) at constant flow rate (0.2 Lh^−1^) and particle dosage (10 g). In the case of the Pb^+2^/Cd^+2^ solutions, q_exp_ exceeded 1.05 and 0.50 mg·g_RWPG_^−1^, respectively, and breakpoint exceeded 110 and 50 BV, respectively; in the case of the Pb^+2^/Ni^+2^ solutions, q_exp_ exceeded 1.20 and 0.30 mg·g_RWPG_^−1^, respectively, and breakpoint exceeded 115 and 30 BV, respectively. The lower capacities obtained after elution of binary solutions respect to single solutions were associated to the metals steric hindrance at the glass surface, with Pb^+2^, Cd^+2^, and Ni^+2^ hydrated radii of 4.01 Å, 4.26, and 4.04 Å, respectively.

**Figure 5:**
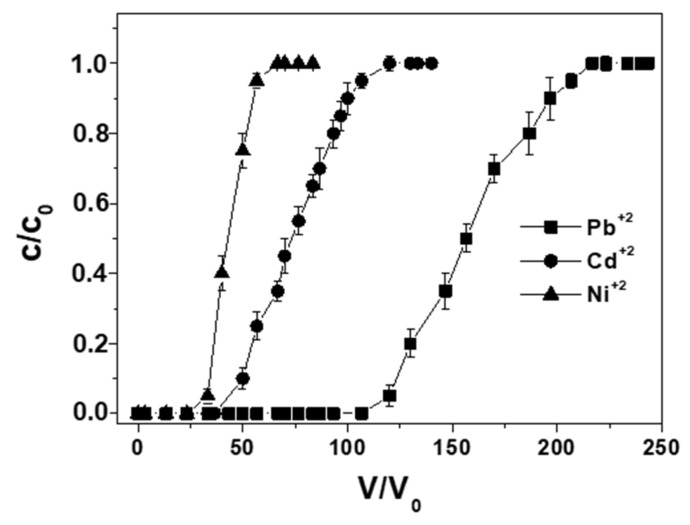
Breakthrough curves in distilled water for multimetal solutions of lead, cadmium, and nickel ions (0.2 Lh^−1^, 2 mg Me^+2^ L^−1^, 10 g RWPG, 0.5–1 mm bead size range, pH = 6, and T = 298 K).

Figure 5 shows a comparison of the breakthrough curves for tests performed in ternary solutions (2 mg Me^+2^ L^−1^) at constant flow rate (0.2 Lh^−1^) and particle dosage (10 g). Overall retention capacities were 1.00, 0.45, and 0.25 mg·g_RWPG_^−1^ and breakpoints were 106, 40, and 20 BV for Pb^+2^, Cd^+2^ and Ni^+2^, respectively (Table 4). 

The lower capacities obtained after elution of ternary metal solutions respect to binary and single ion solutions can be ascribed to the further increase of steric hindrance of the hydrated metal ions at the glass surface. In single, binary, and ternary solutions the lead ion was the most exchanged ion because it was the most interacting specie at the silicate functionalities, while a sensible reduction was observed in the case of cadmium and nickel ions. Specifically, the selectivity of the sorbent towards the metals was partially ascribed to the steric hindrance of the exchanging species but it was also ascribed to the relative free energies of hydration/dehydration of lead (−357.8 Kcal·g-ion^−1^), cadmium (−430.5 Kcal·g-ion^−1^), and nickel (−494.2 Kcal·g-ion^−1^) hydrated ions. Lead ions were the best-sorbed because with the lowest hydrated radius and free energy of hydration, while the lower free energy of hydration of cadmium ions may explain the better retention by RWPG functional groups respect to nickel ions. As showed in previous works [36,59], metal ions and specifically lead ions were mostly retained onto the silicate functional groups of glass by strong Coulomb interactions (ion exchange), and to a lesser extent by Van der Waals weak interactions on other nonspecific RWPG functional groups. In fact, after determination of the ratio between the sorbed lead ion equivalents and the equivalents of the ions released by the glass, it can be concluded that an ion exchange reaction with sodium ions (~75%) present on the glass surface predominantly occurred (Equation (3)) [36]:

2Glass-Na + Me^+2^ → (Glass)_2_-Me +2Na^+^ (3)

**Figure 6:**
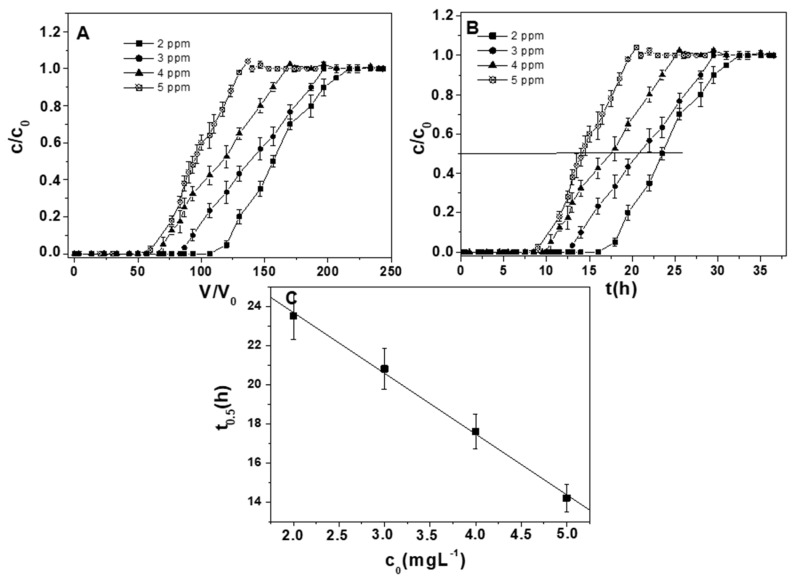
(**A**) Breakthrough curves in distilled water for lead ions in ternary solutions. Measurements carried out with 2, 3, 4, and 5 mg Pb^+2^ L^−1^ (0.2 Lh^−1^ flow rate, 10 g RWPG, 0.5–1 mm bead size range, pH = 6, and T = 298 K). (**B**) Time-dependent breakthrough curves. (**C**) Correlation of the half exchange time, t_0.5_ (C/C_0_ = 0.5 in the breakthrough curves) vs. influent concentration.

Figure 6A and Table 4 show the influence of lead ions concentration (2–5 mg Me^+2^ L^−1^) in multimetal solutions, with constant particle dosage (10 g) and flow rate (0.2 Lh^−1^). A sensible increase of the retention capacities was observed with the increase of the influent concentration. Specifically, retention capacities exceeding 1, 1.2, 1.4, and 1.5 mg·g_RWPG_^−1^ for 2, 3, 4 and 5 mgL^−1^, respectively, were obtained. Moreover, an earlier RWPG exhaustion was detected, i.e., the breakthrough point was reached at lower BV values. In fact, in the case of the most diluted lead ion solution (2 mg L^−1^), the breakpoint was reached at 106 BV as opposed to the 50 BV of the 2.5 times more concentrated solution (5 mg L^−1^) (Table 4) [60,61].

So, as the influent concentration increases the bed sorption capacity increases and higher metal concentrations may saturate the sorbent more quickly, with a consequent reduction of the breakthrough point. 

Lead ions kinetics were obtained by time-dependent breakthrough curves (C/C_0_ vs. t), accordingly, a reverse linear dependence of t_0.5_ vs. C_0_ was observed (Figure 6B,C). This result demonstrated that the mass transfer phenomena and the rate determining step of the process were represented by the film diffusion control (resistance to ion interdiffusion into the liquid–solid Nernst interface) (Equation (2)) [36].

**Figure 7:**
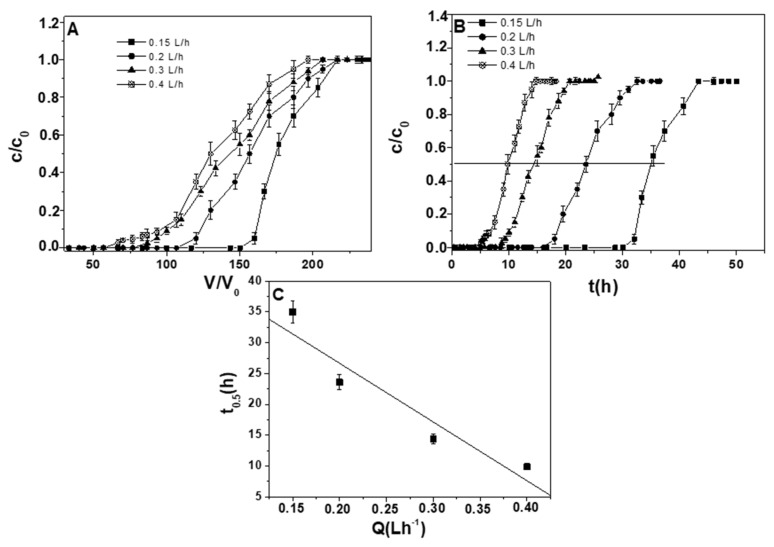
(**A**) Breakthrough curves in distilled water for lead ions in ternary solutions. Measurements were carried out at 0.15, 0.2, 0.3, and 0.4 Lh^−1^ flow rate, 2 mg Pb^+2^ L^−1^, 10 g RWPG, 0.5–1 mm bead size range, pH = 6, and T = 298 K). (**B**) Time-dependent breakthrough curves. (**C**) Correlation of the half exchange time and t_0.5_ (C/C_0_ = 0.5 in the breakthrough curves) vs. flow rate.

Figure 7A and Table 5 show the influence of flow rate (0.15–0.4 Lh^−1^) on the lead ion sorption capacities in the case of ternary solutions with constant metals concentration (2 mg Me^+2^ L^−1^). At slower flow rates, a more exhaustive saturation of the column and a self-sharpening of the curve were observed. In fact, a longer contact time between liquid- and solid-phases affected an increase of the overall capacities and a delay of the breakthrough points. In the case of lead ion solution, values exceeding 1.2, 1.0, 0.9, and 0.80 mg·g_RWPG_^−1^ and breakpoints exceeding 150, 106, 78, and 67 BV were obtained for 0.15 Lh^−1^, 0.2 Lh^−1^, 0.3 Lh^−1^, and 0.4 Lh^−1^, respectively. These results may be explained by the presence of two energetically different interacting functionalities present on the surface of the glass. In the case of a shorter contact time between metal ions and RWPG functional groups, only the more kinetically active functionalities were available for the sorption and a decrease of the overall capacities with anticipated breakthrough points was observed. In the case of a longer contact time between metal ions and RWPG functional groups, all the strong and weak interacting functionalities were readily available for the sorption; this effect induced an increase in the overall capacities and a delay of the breakthrough points [60,61].

Time-dependent lead ion breakthrough curves (C/C_0_ vs. t) allowed for obtaining of the retention kinetics of this metal. At increasing flow rates, an advance of the metal breakthrough was observed because of the shorter contact times. Moreover, an inverse linear dependence of the flow rates vs. t_0.5_ (Figure 7B,C) and consequently a direct linear dependence of the film thickness vs. t_0.5_ were obtained [36]. In fact, at higher flow rates, a thinner Nernst liquid film was formed, while at lower flow rates, a thicker Nernst liquid film around the particles was formed, results which again demonstrated that the rate determining step of the process was represented by the film diffusion control (Equation (2)). 

**Figure 8:**
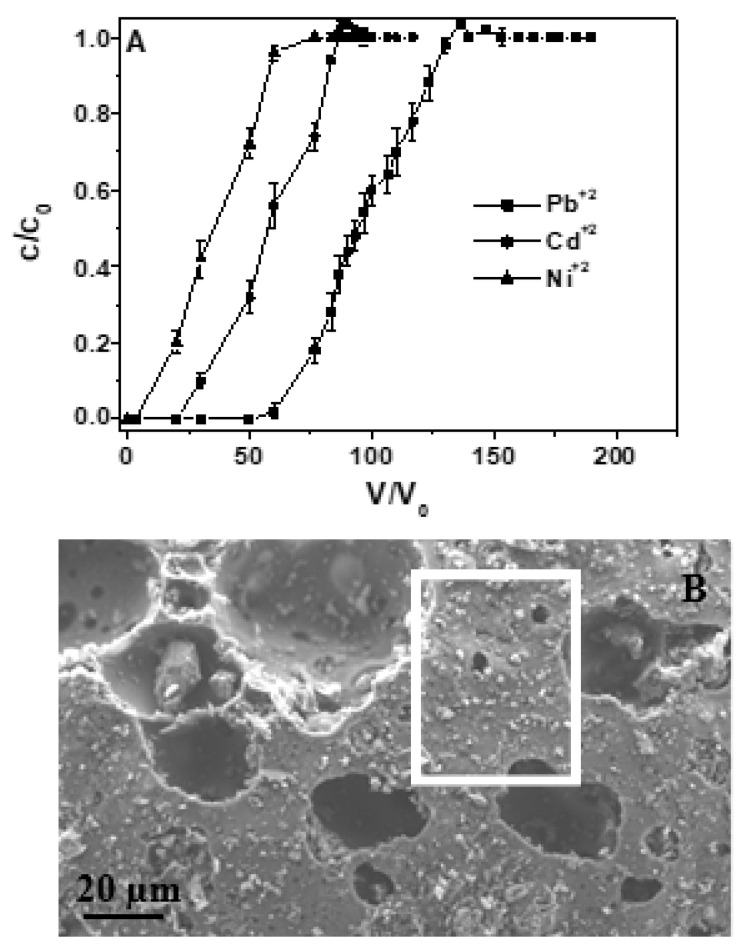
(**A**) Column test carried out in ternary solution (0.5–1 mm RWPG particle size, 10 g RWPG, 0.2 Lh^−1^, 5 mg Me^+2^ L^−1^, pH = 6, and T = 298 K). (**B**) SEM image of a RWPG bead after the sorption process.

After test no. 10, performed at 0.2 Lh^−1^ flow rate and 5 mg Me^+2^ L^−1^ multimetal solution, heavy metal-exhausted RWPG particles were structurally and semiquantitatively characterized. Also in this column experiment, Cd^+2^ and Ni^+2^ capacities were sensibly lower than Pb^+2^ due to the steric hindrance and the relative free energies of hydration/dehydration of the exchanging species (Figure 8A). SEM observations (Figure 8B) revealed that the porous structure of the sorbent did not show any modification while EDX analysis (in Table 6) showed that the metals overall capacity ratios obtained by the breakthrough curves were similar to the wt% ratios between the metal species retained by the glass surface.

In order to minimize the environmental impact of the exhausted sorbent, glass beads were used as aggregates in cement composites and, as demonstrated in previously published papers [59,62], the resulting mortars showed negligible release of metals after jar test [57]. In fact, the concentration of these pollutants in the liquid-phase (distilled water) was below the maximum allowable concentrations for hazardous waste disposal in controlled landfills. Specifically, lead ion concentration was approximately equal to 2 µg L^−1^ (maximum allowable concentration is 10 µg L^−1^), cadmium ion concentration was ~1 µg L^−1^ (maximum allowable concentration is 5 µg L^−1^), and nickel ion concentration was ~1 µg L^−1^ (maximum allowable concentration is 50 µg L^−1^).

**Figure 9:**
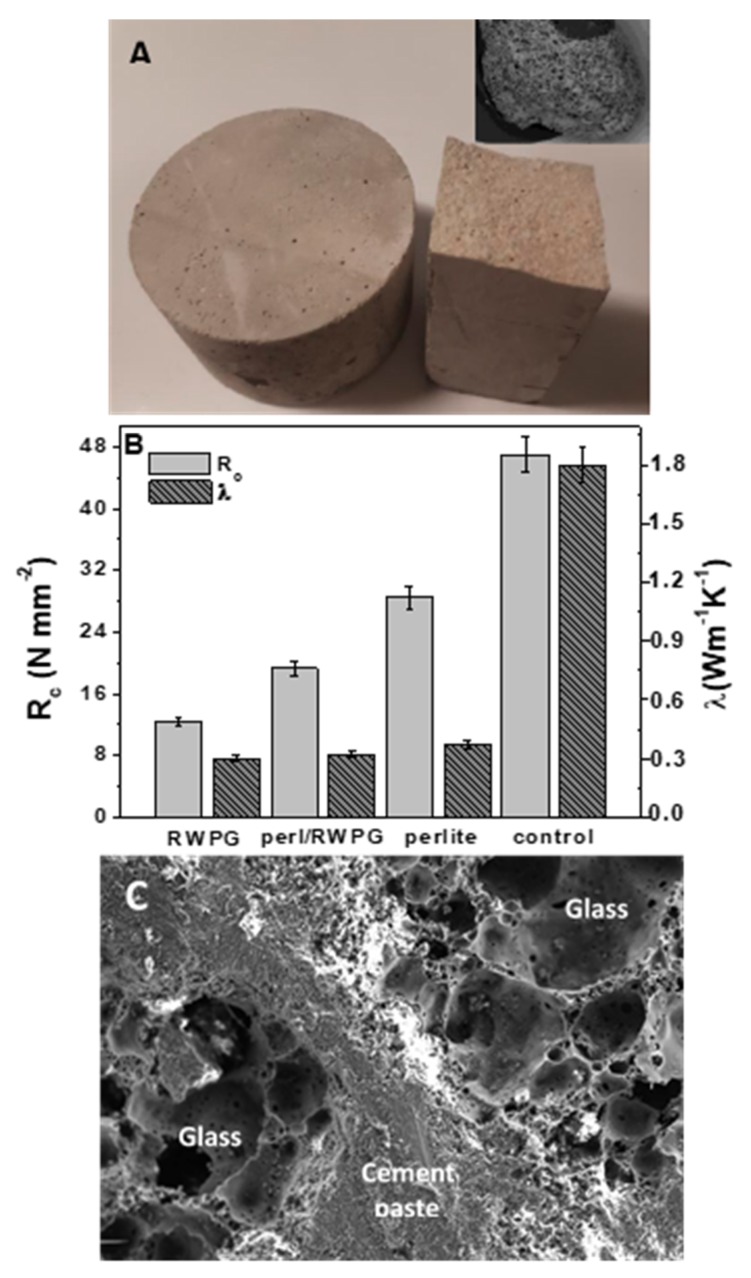
(**A**) Cement mortars with RWPG/perlite mixture as aggregate for thermal measurements (left) and after mechanical tests (right). In the inset: SEM image of a perlite bead. (**B**) Mechanical and thermal tests results for the lightweight mortars and comparison with the sand control. (**C**) SEM image of the cement/RWPG composite.

Afterwards, the lightweight and porous specimens were mechanically and thermally characterized after preparation of prisms and cylinders which were cured for 28 days (Figure 9A). To the purpose, after RWPG/cement specimen preparation (RWPG sample), thermal conductivities exceeding 0.30 Wm^−1^ K^−1^ and compression strengths exceeding 12.4 Nmm^−2^ were observed (Figure 9B). A porous alumosilicate aggregate as perlite (the porous structure is showed in the SEM image of the Figure 9A inset) was added in order to give higher mechanical resistances [63,64] to the conglomerates. As ion exchanger, perlite may be also used after heavy metals exhaustion [65]. Specifically, half RWPG volume was replaced by perlite and it was observed that the new conglomerate (perl/RWPG sample) was equally thermal insulating (0.32 Wm^−1^ K^−1^), whereas the mechanical resistances were higher (19.3 Nmm^−2^). In the case of bare perlite conglomerates (perlite sample), thermal conductivities exceeding 0.37 Wm^−1^ K^−1^ and compression strengths exceeding 28.5 Nmm^−2^ were observed. All of these samples were more thermally insulating with respect to the control which, characterized by normalized sand, was the most compressive-resistant (Figure 9B). A SEM image of the RWPG specimen is shown in Figure 9C where good adhesion of the aggregate to the cement paste was observed, a result which ascribed to the high roughness of the beads and to the similar composition (silicates and aluminates) of glass and cement paste. 

From these results, it can be concluded that these environmental safe conglomerates may be applied in the construction industry as panels or plasters [66,67,68,69,70] because of the lightweight and thermo-insulating properties ascribed to the peculiar features of these aggregates.

## 4. Conclusions

Recycled waste porous glass (RWPG) was used in wastewater treatments for the removal of lead ions from single, binary and multimetal solutions (with cadmium and nickel ions). Experiments were performed in packed bed columns, with a constant bead size range (0.5–1 mm) and constant glass volume and dosage (30 cm^3^ and 10 g), until exhaustion. 

In the case of single and binary solutions, the columns were percolated at 0.2 Lh^−1^ (2 mg Me^+2^ L^−1^); in the case of ternary solutions, the columns were percolated at 0.15–0.4 Lh^−1^ (2 mg Me^+2^ L^−1^), with 2–5 mg Me^+2^ L^−1^ and influent concentration 0.2 Lh^−1^.

Lead ions were mostly retained onto the silicate functional groups of glass by strong Coulomb interactions (ion exchange, ~75%) but also by Van der Waals weak interactions on other nonspecific functionalities, with retention capacities of 1.45 mg·g^−1^ in single solution, 1.05–1.20 mg·g^−1^ in binary solutions, and 0.8–1.5 mg·g^−1^ in multimetal solutions. The selectivity of the sorbent towards lead ions was partially ascribed to the steric hindrance of the hydrated ions but also to the relative metals free energies of hydration/dehydration.The rate determining step of the process was represented by the film diffusion control, i.e., the resistance to ion interdiffusion into the liquid–solid Nernst interface, due to the prevalence of the glass functional groups on the particle surface. 

An increase of the influent lead ion concentration increased the bed sorption capacity (q_exp_) from 1.0 to 1.5 mg·g_RWPG_^−1^ (multimetal solution) with a more quickly saturation of the sorbent and a consequent reduction of the breakthrough point (BV from 106–50). Moreover, at slower flow rates a more exhaustive saturation of the column by lead (from 0.8 to 1.2 mg·g_RWPG_^−1^) and a self-sharpening of the breakthrough curve were observed.

The structure of the sorbent did not show any modification as observed by SEM analysis and EDX characterization which revealed that the Wt.% ratios between the metal species retained by the glass surface were similar to the metals overall capacity ratios. 

In real wastewater the yields of the sorbent may vary, mainly due to the presence of competitive ions. Such interference could decrease the extraction efficiency of the metals; accordingly, these ions may need to be removed as they could be contaminants. For this reason, further research will have to be carried out in order to evaluate the applicability of recycled glass as adsorbent material in real wastewater and to improve the efficiencies of the process after specific treatments of the sorbent.

Glass beads were finally encapsulated in a cement mortar in order to minimize the environmental impact of the exhausted sorbent, in fact negligible leaching of metals was observed. Moreover, the lightweight conglomerates showed very low thermal conductivities (0.3–0.32 Wm^−1^ K^−1^) and discrete mechanical strengths (12–19 Nmm^−2^), for this reason applications in the construction industry as thermal insulating panels or plasters can be proposed.

## Figures and Tables

**Table 1:** Summary of the column tests carried out on recycled waste porous glass (RWPG) sorbent (10 g RWPG, 0.5–1 mm bead size range, pH = 6, and T = 298 K).

Test No.	Metal Specie	Solution	Flow Rate (Lh^−1^)	Influent Concentration (mgL^−1^)
1	Pb^+2^	single	0.2	2
2	Cd^+2^	single	0.2	2
3	Ni^+2^	single	0.2	2
4	Pb^+2^	binary	0.2	2
	Cd^+2^	binary	0.2	2
5	Pb^+2^	binary	0.2	2
	Ni^+2^	binary	0.2	2
6	Pb^+2^	ternary	0.2	2
	Cd^+2^	ternary	0.2	2
	Ni^+2^	ternary	0.2	2
7	Pb^+2^	ternary	0.2	3
8	Pb^+2^	ternary	0.2	4
9	Pb^+2^	ternary	0.2	5
	Cd^+2^	ternary	0.2	5
	Ni^+2^	ternary	0.2	5
10	Pb^+2^	ternary	0.15	2
11	Pb^+2^	ternary	0.3	2
12	Pb^+2^	ternary	0.4	2

**Table 2:** Sorption parameters for single ion solutions of lead, cadmium, and nickel ions (0.2 Lh^−1^ flow rate, 2 mg Me^+2^ L^−1^, 10 g RWPG, 0.5–1 mm bead size range, and T = 298 K).

Test No.	Metal	Solution	q_exp_ (mg·g^−1^)	BV
1	Pb^+2^	single	1.45 ± 0.1	160 ± 8
2	Cd^+2^	single	0.85 ± 0.05	80 ± 4
3	Ni^+2^	single	0.5 ± 0.05	40 ± 2

**Table 3:** Sorption parameters of binary ion solutions of lead, cadmium and nickel ions (0.2 Lh^−1^ flow rate, 2 mg Me^+2^ L^−1^, 10 g RWPG, 0.5–1 mm bead size range, and T = 298 K).

Test No.	Metal	Solution	q_exp_ (mg·g^−1^)	BV
4	Pb^+2^	binary	1.05 ± 0.1	110 ± 6
	Cd^+2^		0.5 ± 0.05	50 ± 3
5	Pb^+2^	binary	1.2 ± 0.1	115 ± 6
	Ni^+2^		0.3 ± 0.0	30 ± 2

**Table 4:** Sorption parameters for multimetal solutions of lead, cadmium and nickel ions at different metal concentration (0.2 Lh^−1^ flow rate, 10 g RWPG, 0.5–1 mm bead size range, and T = 298 K).

Test No.	Metal Specie	Solution	Influent Concentration (mgL^−1^)	q_exp_ (mg·g^−1^)	BV
6	Pb^+2^	ternary	2	1.0 ± 0.05	106 ± 5
	Cd^+2^	ternary	2	0.45 ± 0.0	40 ± 2
	Ni^+2^	ternary	2	0.25 ± 0.0	20 ± 1
7	Pb^+2^	ternary	3	1.2 ± 0.05	83 ± 4
8	Pb^+2^	ternary	4	1.4 ± 0.05	65 ± 3
9	Pb^+2^	ternary	5	1.5 ± 0.05	50 ± 3
	Cd^+2^	ternary	5	0.9 ± 0.05	16 ± 1
	Ni^+2^	ternary	5	0.50 ± 0.0	7 ± 0.5

**Table 5:** Sorption parameters for multimetal solutions of lead, cadmium, and nickel ions at different flow rates. Measurements carried out with 2 mg Me^+2^ L^−1^, 10 g RWPG, 0.5–1 mm bead size range, and T = 298 K.

Test No.	Metal Specie	Solution	Flow Rate (Lh^−1^)	q_exp_ (mg·g^−1^)	BV
10	Pb^+2^	ternary	0.15	1.2 ± 0.05	150 ± 7
7	Pb^+2^	ternary	0.2	1.0 ± 0.05	106 ± 5
11	Pb^+2^	ternary	0.3	0.9 ± 0.05	78 ± 4
12	Pb^+2^	ternary	0.4	0.8 ± 0.05	67 ± 3

**Table 6:** EDX analysis of the metals laden RWPG surface (elemental analysis of the glass surface with the weight percentage (Wt.%) of atoms and relative error).

Element	Wt.%	Wt.% Sigma
Na	11.6	0.25
Al	3.5	0.1
Si	66.0	0.5
Ca	9.8	0.15
Ni	1.5	0.1
Cd	2.4	0.2
Pb	5.2	0.3
Total:	100.00	
